# A Novel Insight into Keratoconus: Mechanical Fatigue of the Cornea

**Published:** 2012

**Authors:** Amir Norouzpour, Alireza Mehdizadeh

**Affiliations:** 1 Eye Research Center, Khatam-Al-Anbia Eye Hospital, Mashhad University of Medical Sciences, Mashhad, Iran; 2 Department of Medical Physics, Shiraz University of Medical Sciences, Shiraz, Iran

**Keywords:** Corneal ectasia, Keratectasia, Keratoconus, Mechanical fatigue

## Abstract

An integrated model for keratectasia risk assessment has received much attention over many years. The objective of this article is to propose a more complete, conceptual model by which high risk individuals can be screened, even with no topographic irregularity or corneal thinning. In this model, corneal ectasia results from the fatigue effect of cyclic shear stress and tensile stress, caused by eye rubbing and fluctuating intraocular pressure (IOP), respectively, on corneal microstructures. The model clarifies the importance of the magnitude of shearing force applied by eye rubbing, the amplitude of IOP fluctuations, the frequency of eye rubbing and IOP fluctuations, the geometry of the cornea, the temperature of the cornea, and the effects of oxidative stress on the cornea in keratectasia development. Therefore, preoperative screening strategies based on these concepts can be designed to assess the risk of keratectasia at an early stage, and select the best candidates who can benefit from keratorefractive surgeries.

## INTRODUCTION

An integrated model for keratoconus (KCN) development has received much attention over many years [1]. Such a model can be widely used for the assessment of keratectasia risk at the preoperative screening stage. Although abnormally low corneal thickness is a strong risk factor for KCN development, the fact that KCN can also occur in individuals with corneal thicknesses >600µm [2] is strong evidence that the present models used for ectasia risk assessment lack certain key indicators of keratectasia. Confusion has arisen in part due to the reliance on late features of biomechanical failure, such as topographic irregularity and corneal thinning, as well as an inability to measure the intrinsic biomechanical characteristics that ultimately give rise to keratectasia. Therefore, this concern demands the efforts of groups to better understand the mechanism of KCN development, and to develop more complete models of ectasia risk assessment based on relevant measures in individual patients. The objective of this article is to propose a more complete, conceptual model for KCN development that incorporates strong biomechanical risk factors of KCN, such as increased intraocular pressure (IOP) and eye rubbing, in order to draw attention to the importance of shear stress between corneal structures. 

Major mechanical stresses which are applied on the cornea include tensile stress (TS) and shear stress (SS) (see figure 1). TS is derived from IOP, with the magnitude of TS being directly proportional to IOP and the radius of the eye, and inversely proportional to the corneal thickness. SS is the tangential force that acts on the unit area of two structures which resist sliding against each other. SS is expressed as force/unit area (N/m² or Pascal [Pa] or dyne/cm²; 1 N/m² = 1 Pa = 10 dyne/cm²).

Diurnal fluctuations of IOP make the applied TS on the cornea cyclic. In addition, SS applied by eye rubbing on the cornea is also cyclic for each episode of rubbing. Cyclic TS and cyclic SS which are applied on the cornea might have mechanical fatigue effects on the corneal structures.

**Figure A:**
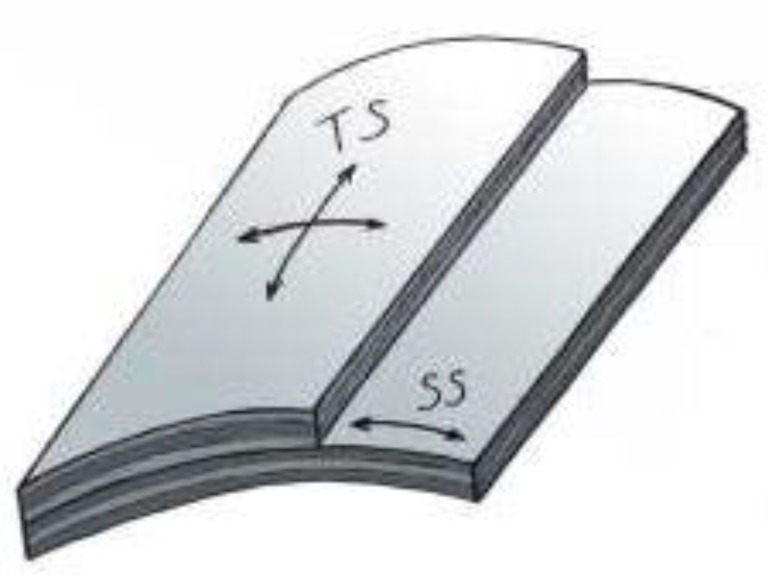
Cross section of cornea showing tensile stress (TS) applied by intraocular hydrostatic pressure and shear stress (SS) between corneal structures resisting against sliding on each other

**Mechanical fatigue**

Fatigue, in materials science, is the progressive and localised structural damage that occurs when a material is subjected to cyclic loading. In this repetitive loading condition, with damage accumulation, fatigue failure (breakage) eventually occurs when stress levels are much lower than those needed to produce failure following a single maximal load (i.e. the maximum stress values are less than the ultimate strength) [3]. Fatigue failure occurs in polymers, metals and other substances. Fatigue life is considered to be the number of cycles before a material fails (i.e. it breaks), and is dependent on different factors, such as the magnitude of cyclic stress and residual stress, the geometry of materials, chemical environmental conditions and temperatures. If the magnitude of cyclic stress increases, the fatigue life of the material decreases and the material breaks after fewer load cycles. Moreover, the geometry of materials determines the stress distribution on them. Stress may be higher in some points, which are called stress-concentration points. For example, the branch areas of pipe joints withstand higher intramural stress than their neighbouring areas [4]. Mechanical fatigue damage is more likely to occur when and where the magnitude of cyclic load is higher. Fatigue damages begin to accumulate at points of stress-concentration until it finally results in fatigue failure. The chemical environment of materials can also influence their structure and their microscopic geometry. It can cause erosion, corrosion, or gas-phase embrittlement, all of which affect the fatigue life.

Unlike materials in which damage is cumulative and materials are not repaired spontaneously when rested, in biologic systems, a tissue turns over continuously with different rates and thereby undergoes continuous remodelling. As a result, damage may be repaired rather than accumulated during a period of time. In addition, a tissue could be adapted to cyclic stress by increasing its ultimate strength to withstand the applied stress and prevent mechanical damage. Since the processes of repair and adaptation are time-based, fatigue life is considered to be the time to failure rather than the number of cycles to failure, and whether fatigue failure occurs or not depends on the rate of fatigue damage accumulation and the rate of tissue repair. The rate of fatigue damage accumulation is dependent on the frequency (number of cycles/period of time) and the magnitude of cyclic stress and the rate of tissue adaptation.

The concept of fatigue failure is applied to the design, manufacture and maintenance of aircraft, bridges, machinery and other objects exposed to cyclic loads. Moreover, in medicine, this concept has formed a field of study in skeletal sciences [5], and cardiovascular sciences [6].

## HYPOTHESES

The cornea undergoes cyclic SS, caused by eye rubbing, and cyclic TS, caused by diurnal fluctuations of IOP whether the eye is rubbed or not. Therefore, it was hypothesised that the corneal integrity may be influenced by the mechanical fatigue effect of cyclic SS, and of cyclic TS leading ultimately to the disruption of corneal microstructures, and eventually to KCN development. The fatigue effect of cyclic stresses on the corneal structures is determined by the magnitude and the frequency of cyclic stresses, the geometry of the cornea and its chemical environment. These factors, as well as the rate of adaptation, determine the rate of fatigue damage accumulation. Whether fatigue failure of the corneal microstructures occurs or not depends on the balance between the resultant rate of damage accumulation and the rate of corneal repair.

Although the relative significance of the fatigue effect of cyclic SS and the fatigue effect of cyclic TS should be compared experimentally, inspirations given by other evidence can provide new insights. During eye development, corneal structures have been well adapted to undergo TS applied by IOP. However, they have not been as well adapted and organised to undergo the SS applied by eye rubbing. Therefore, it seems that the fatigue effect of cyclic SS on the corneal structures is stronger than that of cyclic TS. It is similar to running in a sock which rubs strongly and unsteadily between the sole of the foot and the shoe. Evidence reported in the literature, discussed below, can be helpful to evaluate this model.

## DISCUSSION

As the magnitude of cyclic stress increases, the fatigue life of the cornea decreases, leading to the more rapid development of KCN. This is consistent with clinical data reporting that KCN develops earlier in eyes rubbed vigorously than in those rubbed gently [7]. In addition, the risk of KCN development in eyes which are exposed to larger IOP spikes is increased [8]. 

As the frequency of cyclic stress increases, the rate of damage accumulation increases. Therefore, the cornea does not get the chance to recover in the periods between eye rubbing episodes, or between IOP fluctuations. Thus, the frequency of eye rubbing episodes and of IOP fluctuations are significant factors for KCN development, as reported in the clinical literature [8]. 

The geometry of the cornea influences SS and TS distribution on the corneal structures producing stress-concentration points on the cornea. Such points of the cornea are more vulnerable to cyclic stress. Therefore, the proposed model predicts that corneal geometric changes (i.e. by corneal oedema, or corneal scar) potentiate the fatigue effect of cyclic SS and TS at stress-concentration points. This is consistent with data reporting that eye rubbing which occurs immediately on waking or after contact lens removal may cause greater corneal damage if the cornea is oedematous [9]. Furthermore, scarred regions of the cornea are more vulnerable to being affected by cyclic stresses [9]. 

The chemical environment of the cornea also influences the fatigue life, which is analogous to the different microscopic structures of materials in different chemical environments. Therefore, ultraviolet radiation and oxidative stress can influence the microstructure of the cornea [10,11] leading to the production of stress-concentration points on the cornea.

In addition, when the cornea becomes warmer, the geometry of the microstructures of the cornea may change, making it more vulnerable to the fatigue effects of cyclic stresses. During prolonged vigorous rubbing, any friction between the palpebral conjunctiva and the cornea, various degrees of hyperaemia induced by rubbing, and eye closure may all raise the corneal temperature leading to an increased risk of KCN development [9]. 

## CONCLUSION

Keratoconus\keratectasia results from the mechanical fatigue effects of cyclic shear stress and tensile stress, caused by eye rubbing and fluctuating IOP, respectively, which are potentiated by the geometry and chemical environment of the cornea. 

Based on these concepts, computational and experimental models can be designed to prevent keratoconus development or progression in high risk individuals. Furthermore, more reliable, preoperative screening strategies can be designed to assess the risk of keratectasia at the preoperative stage, and select the best candidates who may benefit from keratorefractive surgeries.

## DISCLOSURE

The authors report no conflicts of interest in this work.
